# Polycystic ovary syndrome: What more can be done for patients?

**DOI:** 10.1016/j.lanepe.2022.100524

**Published:** 2022-10-03

**Authors:** The Lancet Regional Health – Europe

Polycystic ovary syndrome (PCOS) is one of the most common endocrine conditions in women of reproductive age and yet the disorder has remained an enigma for many decades, with little progress made towards the improvement of symptoms and wellbeing of patients. The global prevalence of PCOS varies from 5 to 18%, with an average prevalence of 276·4 cases per 100 000 people in Europe. Around 50% of women are not aware that they have PCOS or they have a delayed diagnosis. Considering the high prevalence and underdiagnosis of PCOS, it is now more important than ever to reassess the management and needs of patients with the condition.

One main reason for the lack of effective treatments for PCOS is that the causes are complex and multifactorial, including genetic and epigenetic susceptibility, hypothalamic and ovarian dysfunction, excess androgen exposure, insulin resistance, and adiposity-related mechanisms. Common symptoms include ovulatory and menstrual dysfunction, hirsutism, and weight gain. Metabolic abnormalities—mainly insulin resistance—are evident, especially among individuals who also have hyperandrogenism (excessive presence of testosterone). No clear diagnostic criteria exist, but a diagnosis can usually be made if the patient meets at least two of the following Rotterdam criteria: hyperandrogenism, irregular menstrual cycles, and polycystic ovary morphology.

PCOS is also strongly associated with other long-term health risks including type 2 diabetes, endometrial cancer, and cardiovascular disease; therefore, more needs to be done for women with PCOS to improve their quality of life and prevent the known associated risks. In addition to the long-term health risks, the psychological wellbeing of patients with PCOS is an important consideration. A 2021 study found that 40% of women with PCOS have depression and 16·6% have mood disorders, indicating that at least 56·6% of women with the condition have mental health problems. Causes can include low confidence as a result of hirsutism and managing the side-effects of the contraceptive pill, which is usually prescribed to treat the symptoms. Furthermore, the psychological effects for women of reproductive age are substantial and can lead to psychosocial distress due to fertility problems. Women with PCOS are not only left behind medically but are also often less motivated to self-manage the condition over time. Furthermore, adolescents and young women might experience bullying and trolling in response to their hirsutism, which might further negatively affect mental health, resulting in low self-confidence and body image issues.

PCOS is difficult to manage both for health-care professionals and patients as different phenotypes exist and management is dependent on an individual's symptoms. Prescribing the contraceptive pill to regulate periods, giving general (and sometimes, vague) advice on how to self-manage symptoms such as acne and hirsutism, and ways to self-improve diet and lifestyle, are not enough. For women trying to get pregnant, clomifene—used to treat infertility in women who do not ovulate—is usually recommended, and metformin—often used to treat type 2 diabetes—can be recommended to lower the level of insulin; however, these drugs have not been approved by medical regulatory authorities for PCOS per se, since they are symptom specific and are not disease modifying drugs. The SPIOMET4HEALTH trial being conducted in numerous European countries is a step forward, which aims to produce a single tablet that combines three medicines that have already been proven to be effective for women with PCOS, which would not only improve medicine adherence and quality of life, but would also save €0·5–1 billion per year for health-care systems. It is now time for other global health systems to not only adopt this vital step, but to also think of alternative methods to manage and treat women with PCOS.

With the cost of living increasing, many women with PCOS might not be able to cover costs of laser hair removal or electrolysis to remove hair, gym memberships to reduce weight, and in some countries—especially low-income and middle-income countries—the contraceptive pill to regulate periods. Laser hair removal and electrolysis are not covered by the National Health Service (NHS) England because they are considered cosmetic procedures and funding is only provided for exceptional circumstances, yet NHS Scotland has taken action to make it available.

Small changes, such as providing free access to hair removal services and perhaps to a dietician (in person or via telephone) and adopting accessible ways in which help can be provided, would enable more women to improve their quality of life. More options are needed than currently available, including more funding for research on this topic and approved drugs for use.

At present, there is no cure for PCOS, and this has been known for many decades. It is now time for change. Action is needed by policy makers, health-care systems, and medical regulatory authorities worldwide—not only in Europe and by NHS Scotland—to realise this change and help women with PCOS.

